# Extracellular vesicles from bone marrow-derived multipotent mesenchymal stromal cells regulate inflammation and enhance tendon healing

**DOI:** 10.1186/s12967-019-1960-x

**Published:** 2019-06-25

**Authors:** Zhengzhou Shi, Qi Wang, Dapeng Jiang

**Affiliations:** Department of Urology, Shanghai Children’s Medical Center, Shanghai Jiao Tong University School of Medicine, Shanghai, 200127 China

**Keywords:** Mesenchymal stem cells, Extracellular vesicles, Macrophages, Tendon stem cells, Tendon healing

## Abstract

**Background:**

Extracellular vesicles from bone marrow-derived multipotent mesenchymal stromal cells (BMSC-EVs) can play important roles in the repair of injured tissues. However, no reports have investigated the role and underlying mechanisms of BMSCs-EVs in the tendon repair process. We hypothesized that BMSC-EVs may play a role in modulating inflammation during tendon healing and improving tendon repair in a rat model of patellar tendon injury.

**Methods:**

First, we created window defects in the patellar tendons of Sprague–Dawley rats. Rats (n = 16) were then randomly assigned to three groups: BMSC-EVs group, Fibrin group, and control group. Rats in the BMSC-EVs group were treated with BMSC-EVs and fibrin glue (25 µg in 10 µL). Rats in the fibrin group were treated with fibrin only, and those in the control group received no treatment. Histopathology, immunohistochemistry, and gene expression analyses were performed at 2 and 4 weeks after surgery.

**Results:**

At 4 weeks, tendons treated with BMSC-EVs showed regularly aligned and compact collagen fibers as compared with the disrupted scar-like healing in rats in the fibrin and control groups. The expression of genes related to tendon matrix formation and tenogenic differentiation: collagen (COL)-1a1, scleraxis (SCX), and tenomodulin (TNMD) was significantly higher in the BMSC-EVs group than in the other two groups. With histopathology, we observed significantly higher numbers of CD146+ tendon stem cells and fewer numbers of apoptotic cells and C–C chemokine receptor type 7 (CCR7)-positive proinflammatory macrophages in the BMSC-EVs group. BMSC-EVs treatment also led to an increase in the expression of anti-inflammatory mediators (IL-10 and IL-4) at 2 weeks after surgery.

**Conclusions:**

Overall, our findings show that the local administration of BMSC-EVs promotes tendon healing by suppressing inflammation and apoptotic cell accumulation and increasing the proportion of tendon-resident stem/progenitor cells. These findings provide a basis for the potential clinical use of BMSC-EVs in tendon repair.

## Background

Tendons play key roles in connecting muscles to bones. They are frequently injured in both occupational and athletic activities. Tendon injuries are an acute health care burden and have become a significant challenge in orthopedics [1]. Natural tendon healing is a complex process consisting of three overlapping stages: inflammation, proliferation, and remodeling. Tendon healing is slow compared with other types of connective tissue healing because of its poor vascularization and the excessive load-bearing it must withstand [2]. A previous study showed that inflammatory factors are dramatically upregulated within the first 7 days after tendon injury [3]. An intense early inflammatory cascade often results in the formation of a scar-like tendon, and is associated with chronic matrix degradation and the formation of adhesions, which impede the intrinsic repair process and lead to a tendon with poor tissue quality and inferior mechanical properties [4]. In recent years, tissue engineering and other biological-based therapeutic strategies have been used to modulate inflammation at the repair site to effectively enhance tendon healing [5–7].

The use of mesenchymal stem cells (MSCs) is currently an attractive solution for tendon repair and regeneration. Recent findings show that adipose tissue-derived mesenchymal stromal cells and tendon stem/progenitor cells (TSCs) can modulate the inflammatory environment by regulating the activities of resident macrophages to enhance tendon healing [8, 9]. Indeed, one study showed that bone marrow-derived multipotent mesenchymal stromal cells (BMSCs) can improve early tendon healing both histologically and biomechanically [10]. However, the implantation of BMSCs can also result in ectopic bone formation in the tendon or contribute to teratoma formation [11, 12]. Recent studies have demonstrated that MSC transplantation therapy promotes tissue repair mainly through a paracrine mechanism [13–15], and that EVs play an important role in the function of MSCs [16, 17]. More recently published results indicate that EVs derived from BMSCs (BMSCs-EVs) have promise in attenuating inflammation and apoptosis during tissue repair [18].

We hypothesized that BMSC-EVs may also play a role in modulating inflammation during tendon healing. To test this hypothesis, EVs were isolated from BMSCs and delivered to a rat model of tendon repair. The impact of BMSC-EVs on the expression of proinflammatory cytokines, anti-inflammatory mediators, and macrophages during tendon healing was investigated in vivo. The effect of BMSC-EVs on the quality of the repaired tendon tissue was also characterized.

## Methods

### Isolation and culture of rat BMSCs

Bone marrow-derived multipotent mesenchymal stromal cells were isolated from eight Sprague–Dawley rats. Animal experiments were performed according to the Rules and Regulations of the Animal Care and Use Committee at our University. BMSCs were isolated from rat bone marrow as described previously [10]. Briefly, bone marrow was flushed from the bone marrow cavities, collected into centrifuge tubes, and mononuclear cells were isolated by Ficoll density gradient. The mononuclear cells were then suspended in alpha-modified Eagle’s medium (α-MEM) containing 10% fetal bovine serum (FBS) and plated into T-75 flasks. Cells were incubated at 37 °C with 5% CO_2_, with the medium changed every 3 days. Nonadherent cells were discarded after 48 h. The bone marrow cells at the third passage were harvested and characterized. BMSCs were identified by flow cytometry with a fluorescence-labeled antibody for the positive surface markers CD44 and CD90. The negative surface markers: CD11b and CD34 were investigated as negative marker to exclude hematopoietic lineages. Moreover, morphology and multipotency of BMSCs were measured. The BMSCs used in this study were between passage 3 and 5.

### Isolation and identification of BMSCs-EVs

At 80% to 90% confluence, BMSCs were rinsed with phosphate-buffered saline (PBS) and cultured in Mesen Gro MSC medium (Thermo Fisher Scientific) for an additional 48 h. Conditioned media was collected and EVs were isolated, as described previously [16]. The purification of BMSCs-EVs involves several centrifugation and ultracentrifugation. The conditioned media was centrifuged sequentially at 300×*g* for 10 min followed by 2000×*g* for 10 min to remove cellular debris. The supernatants were then ultracentrifuged at 100,000×*g* for 2 h to obtain a pellet containing the EVs, which was resuspended in 200 μL of PBS. EVs-enriched fraction was centrifuged at 1500*g*, 30 min with 100-kDa molecular weight cutoff (MWCO) hollow fiber membrane (Millipore, Billerica, MA, USA). Then, EVs were passed through a 0.22-μm filter. The total protein concentration in the EVs was quantitated using the Micro Bicinchoninic Acid (BCA) Protein Assay Kit (Pierce) following the manufacturer’s instructions. Transmission electron microscopy (TEM) and western blotting were used to examine the morphology and the quality of the EVs. The size of EVs were analyzed with use of qNano. EVs were stored at 4 °C for no more than 1 h before used for experiments.

### Surgical procedure and treatment

**Fig. 1:**
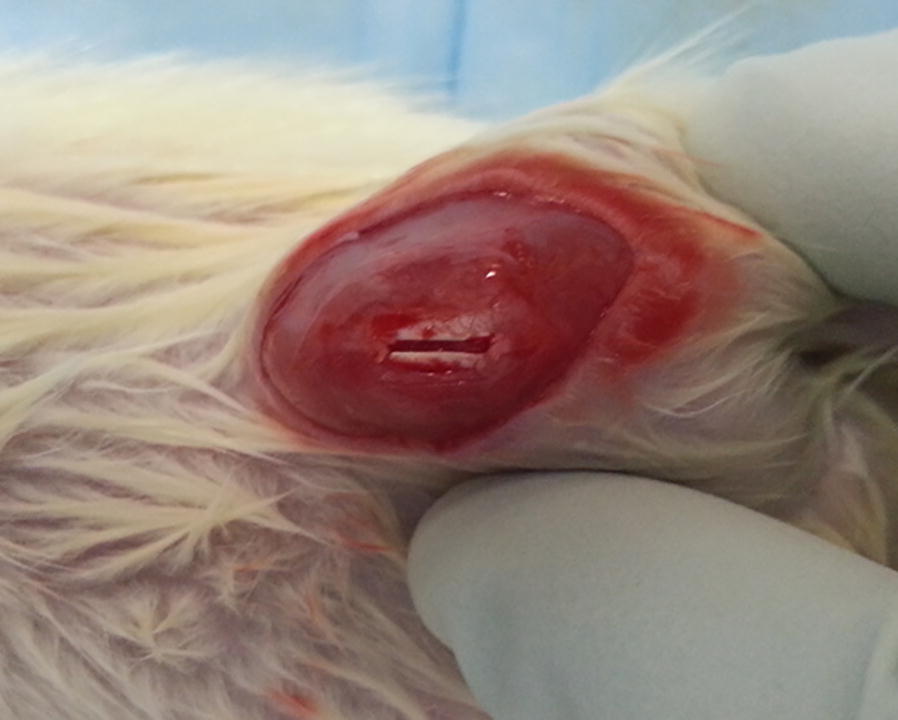
Patellar tendon injury model. The central one-third of the patellar tendon was removed to create a tendon defect

A patellar tendon injury model was created in Sprague–Dawley rats, according to a previously published method [7]. Briefly, in 48 rats, the central one-third of the patellar tendon was removed from the distal apex of the patellar to the insertion of the tibial tuberosity to create a tendon defect (Fig. 1). Then, rats were allocated to one of three groups (*n *= 16): BMSC-EVs group, treated with 10 µL of fibrin containing 25 µg BMSC-EVs; Fibrin group, treated with 10 µL of fibrin glue alone; and the control group, which was left untreated. Fibrin glue containing EVs was placed in the window defect of patellar tendon after rat tendon injury model. The fibrin glue (Baxter^®^, Vienna, Austria) was considered to act as a useful vehicle of growth factors or cells. It has been used extensively in all kinds of surgery and research [19, 20]. Fibrin solution consists of two main components: fibrinogen and thrombin. The final concentration of fibrinogen and thrombin was 80 mg/mL and 600 units/mL, respectively. It could produce a fibrin clot in about 10 s after applied to injury site. Mixing EVs lysates (25 µg) in fibrin glue (10 µL) is easy to deliver locally in window defect of patellar tendon.

At 2 and 4 weeks after surgery, eight rats in each group were killed, and the tissues surrounding the injured patellar tendons were harvested for histology, immunohistochemistry, and gene expression analysis.

### Histology and immunohistochemistry

Rat patellar tendon tissues were harvested at 4 weeks and fixed in 4% formaldehyde solution and embedded in paraffin. Samples were sectioned longitudinally at a thickness of 5 μm, and then processed for histological examination using hematoxylin–eosin (H&E) and Masson’s trichrome staining. The organization of fibrous connective tissue within the defect site was evaluated using a parallel fiber alignment scoring method [21], as previously described: 3 = 75% to 100% parallel fiber alignment; 2 = 50% to 75% parallel fiber alignment; 1 = 25% to 50% parallel fiber alignment; and 0 = 0% to 25% parallel fiber alignment. All sections were analyzed by a single pathologist, who was blinded of the treatment groups.

Specimens harvested at 2 weeks were used for immunohistochemistry to examine expression changes in CD146, cleaved caspase-3, CD163, C–C chemokine receptor type 7 (CCR7), interleukin (IL)-10, and IL-6 (all from Abcam; 1:100 dilution). Immunohistochemical staining with anti-rat type I collagen antibody (1:300; #sc-25974, Santa Cruz Biotechnology, Dallas, TX) was also performed to evaluate tendon healing. Briefly, nonspecific reactive sites were masked with 5% bovine serum albumin before the slides were incubated overnight at 4 °C with primary antibodies at 1:100 or 1:300 dilution, as specified. Slides were then incubated with a species appropriate secondary antibody in the dark at room temperature for 60 min. Nuclei were counterstained with Hoechst fluorochrome 33342 (1 mg/mL; Sigma-Aldrich, St. Louis, MO, #B2261). Positive cells in the healing tendon tissue were quantified using light microscopy at the magnification of 400×, defined by an ocular morphometric grid. For each animal, 15 randomly selected tissue sections were analyzed.

### Gene expression

Gene expression changes in the healing tendons were determined using real time PCR. Rats were anesthetized with isoflurane gas on day 14 after surgery. 8 healing patellar tendon tissues in each group were processed for mRNA extraction. Total RNA was extracted using an RNeasy mini kit (Qiagen; Hilden, Germany). cDNA was synthesized using the First-strand kit (Invitrogen; Carlsbad, CA). qRT-PCR was carried out with the QuantiTect SYBR Green RT-PCR kit (Qiagen). Total RNA isolation, cDNA synthesis, and gene expression assay were performed as described previously [22]. Relative gene expression levels were calculated with the 2^ΔCT^ formula. The gene expression levels were normalized with respect to the control group. Each gene analysis was performed in triplicate.

Rat-specific primers were synthesized by Invitrogen (Carlsbad, CA) and used for tenomodulin (TNMD), scleraxis (SCX), collagen type I, collagen type III, IL-1B, IL-6, interferon gamma (IFNγ), IL-4, IL-10, IL-13, CCR7, and CD163 testing. Glyceraldehyde-3-phosphate dehydrogenase (GAPDH) was used as an internal control (Table 1).

**Table 1:** Rat primers used for qRT-PCR analysis

Gene	Primer sequence
TNMD	F: 5′-CCATGCTGGATGAGAGAGGTTAC-3′ R: 5′-CACAGACCCTGCGGCAGTA-3′
SCX	F: 5′-AACACGGCCTTCACTGCGCTG-3′ R: 5′-CAGTAGCACGTTGCCCAGGTG-3′
COL1a1	F: 5′-CCGGACTGTGAGGTTAGGAT-3′ R: 5′-AACCCAAAGGACCCAAATAC-3′
COL3a1	F: 5′-AACGGAGCTCCTGGCCCCAT-3′ R: 5′-ATTGCCTCGAGCACCTGCGG-3′
IL-1B	F: 5′-AGCAGCTTTCGACAGTGAGG-3′ R: 5′-CTCCACGGGCAAGACATAGG-3′
IL-6	F: 5′-AGAAAAGAGTTGTGCAATGGCA-3′ R: 5′-GGCAAATTTCCTGGTTATATCC-3′
IFN-γ	F: 5′-AGGCCATCAGCAACAACATAAGTG-3′ R: 5′-GACAGCTTTGTGCTGGATCTGTG-3′
IL-10	F: 5′-GGACTTTAAGGGTTACTTGGG-3′ R: 5′- AGAAATCGATGACAGCGTCG-3′
IL-4	F: 5′-GAACTCACTGAGAAGCTGCA-3′ R: 5′-GAAGTGCAGGACTGCAAGT-3′
IL-13	F: 5′-AGACCAGAAGACTTCCCTGT-3′ R: 5′-TCAATATCCTCTGGGTCCTG-3′
CCR7	F: 5′-TGGTCATTTTCCAGGTGTGCT-3′ R: 5′-TACAGGGTGTAGTCCACGGT-3′
CD163	F: 5′-GTAGTAGTCATTCAACCCTCAC-3′ R: 5′-CGGCTTACAGTTTCCTCAAG-3′

### Isolation and culture of rat tendon cells

Patellar tendons from SD male rats were used as explants for primary cell cultures. Patellar tendons were cut into small sections, and then placed and cultured in 6-well culture plates in aseptic conditions. After 5 min of air-drying for better adherence, Dulbecco’s Modified Eagle Medium containing 20% fetal bovine serum and 1% penicillin/streptomycin were supplemented to each well. After 3–4 days in culture, cells began to emerge from the tendon pieces. After the cells reached 80% confluence, tendon cells were detached with trypsin and mixed together as passage 0. Tendon cells at passages 3 were used in the following experiments.

### Collagen type I expression of tendon cells

After culture in media containing BMSC-EVs (0, 10, 20 μg/mL) for 48 h, tendon cells were harvested for RNA extraction. Total RNA was extracted using an RNeasy Mini Kit (Qiagen, Hilden, Germany) according to the manufacturer’s instructions. cDNA was synthesized using a First-Strand Kit (Invitrogen, Carlsbad, CA). qRT-PCR was carried out with QuantiTect a SYBR Green RT-PCR Kit (Qiagen, Hilden, Germany). Gene expression assay were performed as described previously. Glyceraldehyde-3-phosphate dehydrogenase (GAPDH) was used as an internal control. Relative gene expression was calculated using the 2^ΔCT^ formula. Rat-specific primer for collagen type I was as follows: 5ʹ-CCGGACTGTGAGGTTAGGAT-3ʹ (forward) and 5ʹ-AACCCAAAGGACCCAAATAC-3ʹ (reverse).

### Cell proliferation assay

The effects of BMSC-EVs on the proliferation of tendon cells were determined by using the CCK8 assay. Briefly, tendon cells were seeded at 1 × 10^4^ cells/well onto 96-well plates in 200 μL of complete culture medium. After 24 h in culture, cells were treated with medium containing different concentrations of BMSC-EVs (0, 10, 20 μg/mL) for 3 days. The value of optical density was measured with a microplate reader at 450 nm according to the manufacturer’s instructions.

### Statistical analysis

All values are expressed as the mean ± standard deviation (SD). Data were compared by an analysis of variance (ANOVA) followed by Tukey’s test. Because the number of rats was small for parametric statistics, the Kruskal–Wallis test was used to compare the results of histological analysis among groups. Statistical analyses were carried out with SPSS 11.0 statistical package. All *p* values less than 0.05 were considered statistically significant.

## Results

### Characterization of BMSCs and BMSCs-EVs

**Fig. 2:**
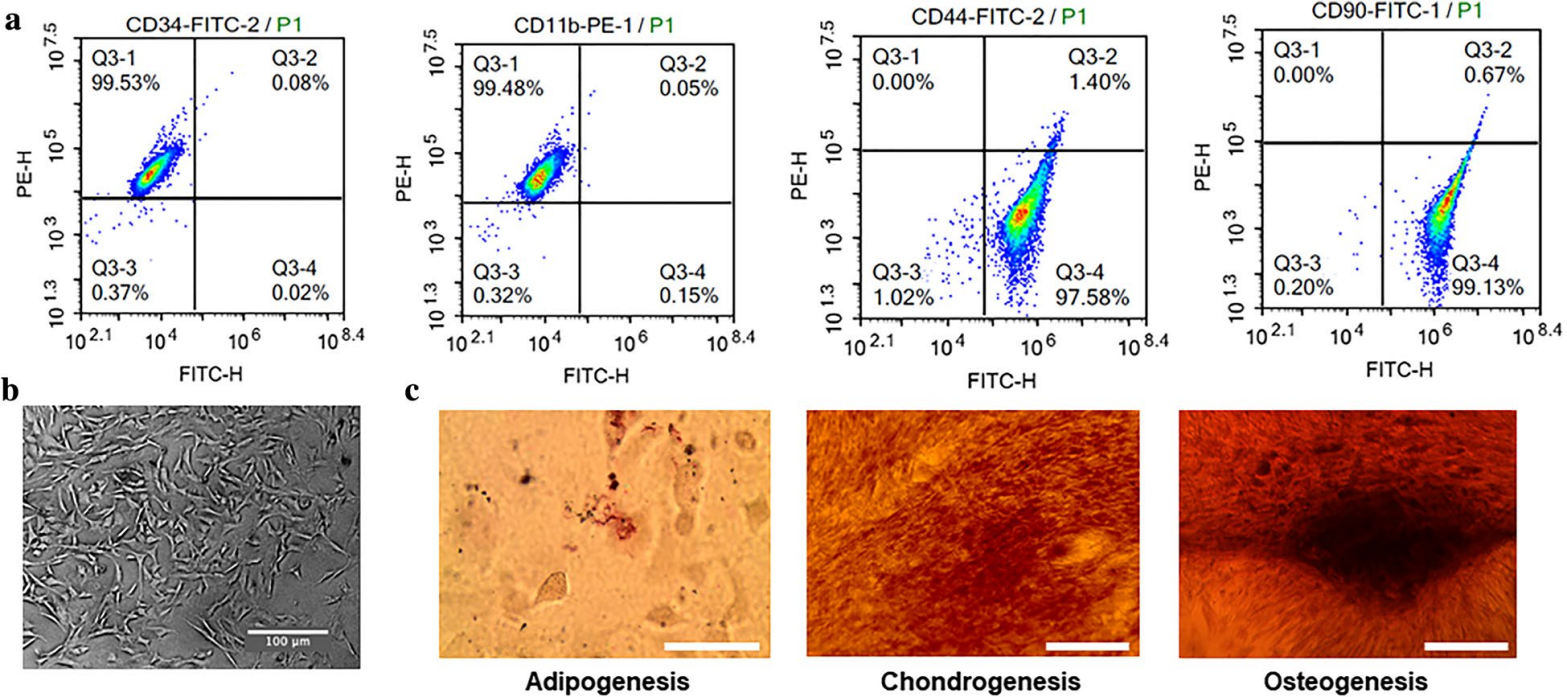
Characterization of BMSCs. **a** FACS analysis for detection of BMSCs surface markers. **b** Morphology and multipotency of BMSCs. Scale bar: 100 μm. **c** The adipogenic, chondrogenic, and osteogenic differentiation potentials of BMSCs. Scale bar: 100 μm

Bone marrow-derived multipotent mesenchymal stromal cells were analyzed for expression of a panel of cell surface markers as shown in Fig. 2. In the FACS analysis, BMSCs were positive for mesenchymal markers CD90 (99.13%) and CD44 (97.58%), but negative for hematopoietic markers CD34 (0.02%) and C11b (0.15%). Morphology and multipotency of BMSCs were showed in Fig. 2.

**Fig. 3:**
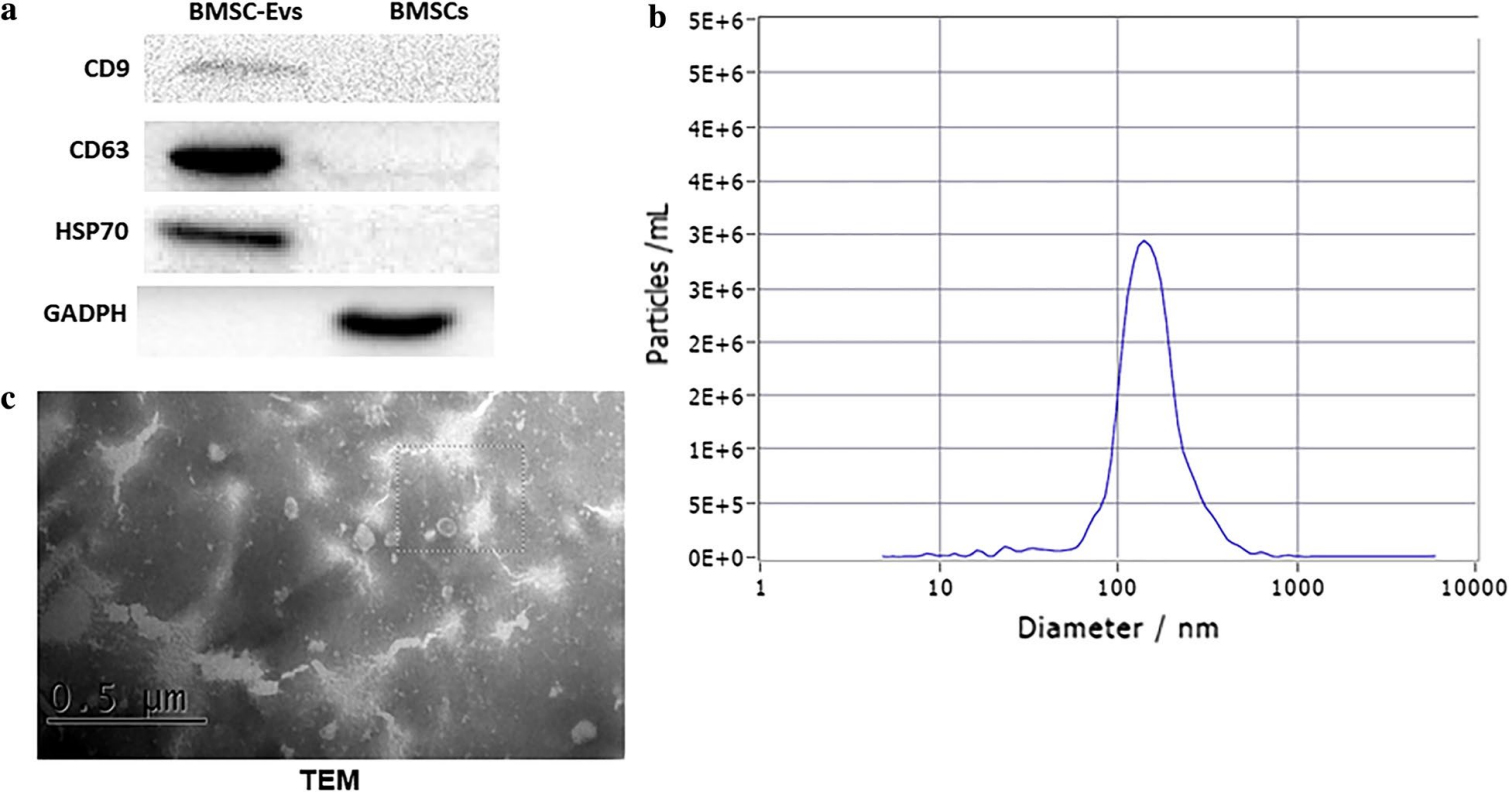
Characterization of BMSC-EVs. **a** Western-Blot was used to confirm the expression of EVs signature markers. **b** Particle size distribution. **c** Morphology using transmission electron microscopy. Scale bar: 0.5 μm

Surface markers CD9, CD63, and HSP70 were presented high expression in BMSCs-EVs (Fig. 3a). Under transmission electron microscopy, BMSCs-EVs appeared as circular particles (Fig. 3c).

### Histological analysis

**Fig. 4:**
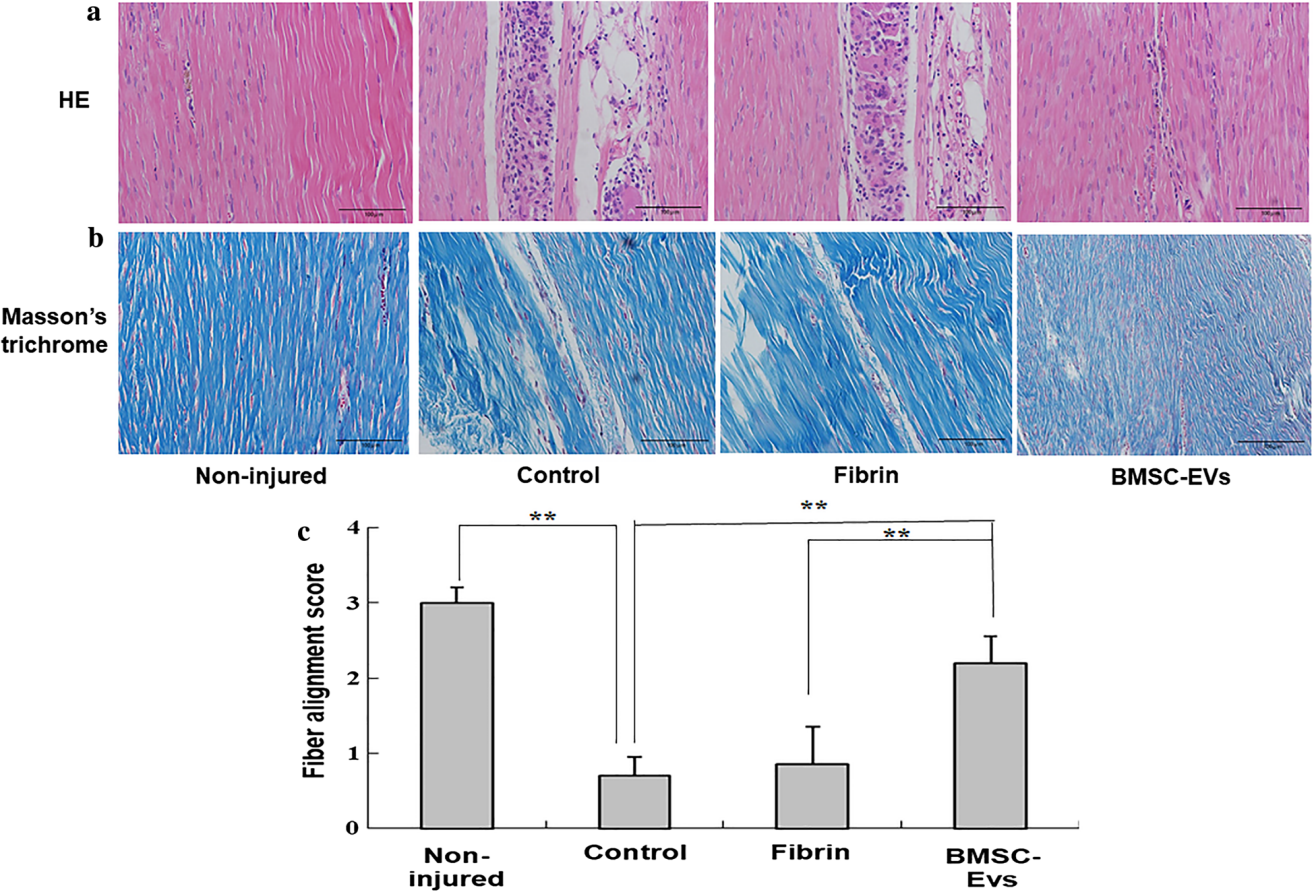
The results of histological evaluation for tendon repair at 4 weeks after surgery. **a** The HE staining of window defect on the patellar tendon at 4 weeks after surgery. **b** Masson’s trichrome staining of window defect on the patellar tendon at 4 weeks after surgery. **c** Fiber alignment score was significantly higher for the rats in the BMSC-EVs and fibrin groups compared with control group (n = 8 donors). Bars: 100 μm, ×20. Data are represented as mean ± SD. **p < 0.01

Tendons treated with BMSC-EVs showed regularly aligned and compact collagen fibers in contrast to the disorganized and disrupted scar-like healing in tendons from fibrin and control groups (Fig. 4a, b). The fiber alignment score was significantly higher for the rats in the BMSC-EVs group compared with those in the fibrin and control groups (Fig. 4c).

### Effect of BMSC-EVs on macrophage polarization and inflammatory response

**Fig. 5:**
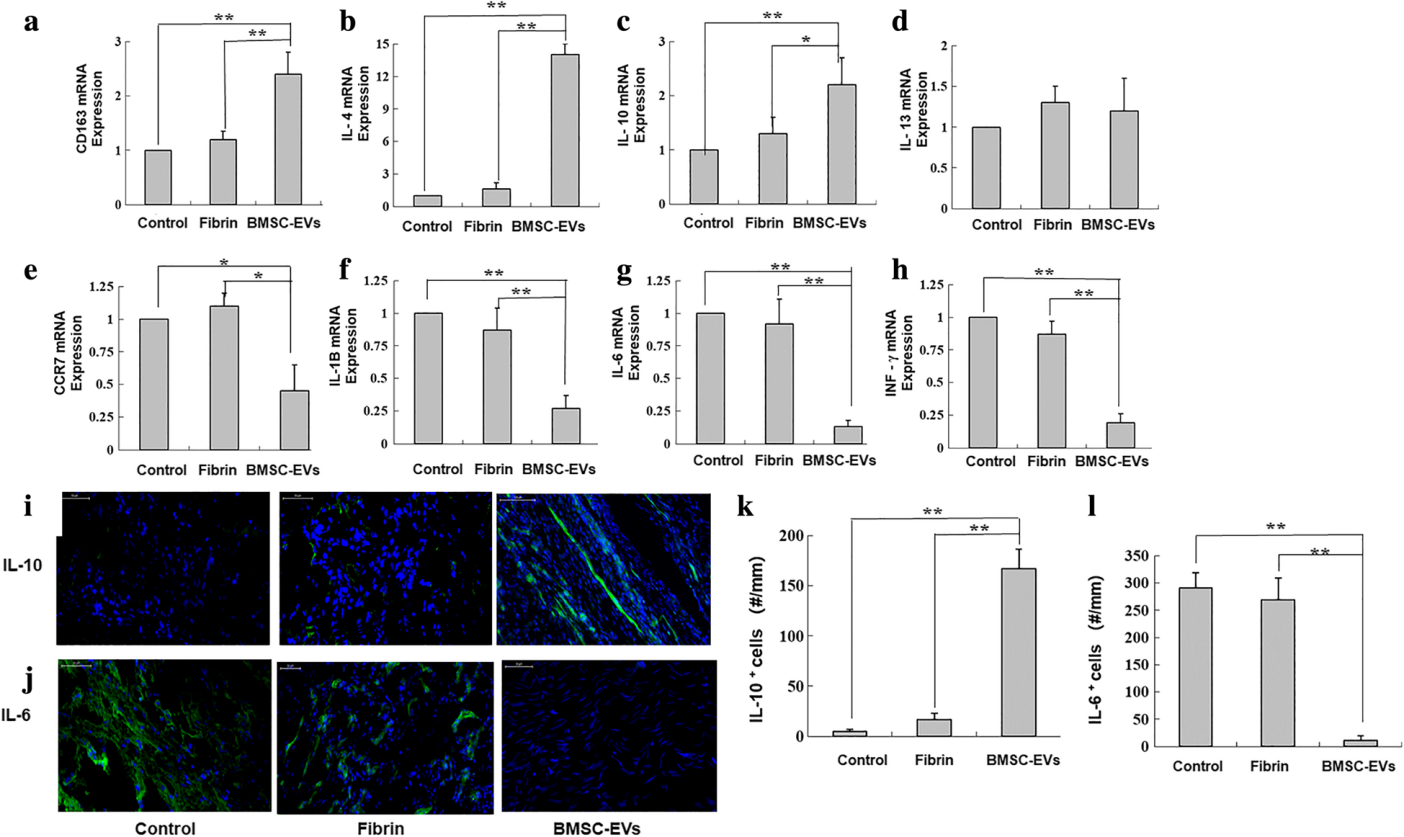
The impact of BMSC-Exos on tendon inflammatory response. The graphs show changes in the expression of **a** CD163, **b** IL-4, **c** IL-10, **d** IL-13, **e** CCR7, **f** IL-1B, **g** IL-6, and **h** INF-γ genes in the repaired tendons (n = 8 donors). The expressions of **i** IL-10+ cells and **j** IL-6+ cells at the repair site were evaluated by immunofluorescence assay. **k** Numbers of IL-10+ cells at the repair site (n = 8 donors). **l** Numbers of IL-6+ cells at the repair site (n = 8 donors). Bars: 50 μm, ×200. Data are represented as mean ± SD. *p < 0.05, **p < 0.01

We found elevated expression of CD163 as a marker of anti-inflammatory macrophages in BMSC-EVs group as compared with the other groups (Fig. 5a). Corroborating this, we found a significant increase in the mRNA expression levels of IL-4 and IL-10 (M2 macrophage stimulator) in the BMSC-EVs group as compared with the fibrin and control groups (Fig. 5b, c). However, there was no significant increase in IL-13 mRNA expression in BMSC-EVs group (Fig. 5d). Furthermore, we found a reduction in the expression in each of IFNγ, IL-1B, IL-6 (markers of M1 macrophages) in the BMSC-EVs group as compared with the fibrin and control groups (Fig. 5f–h).

We also investigated the in vivo inflammatory responses among the three groups. At 2 weeks, we found a significantly higher number of cells were expressing IL-10 in the BMSC-EVs group as compared with the control group (Fig. 5i). Comparatively, abundant IL-6 expression was observed in the control and fibrin tendons (Fig. 5j). These results were confirmed quantitatively, with a significantly higher density of IL-10+ cells and fewer IL-6+ cells in the BMSC-EVs group than in the control group (Fig. 5k, l).

**Fig. 6:**
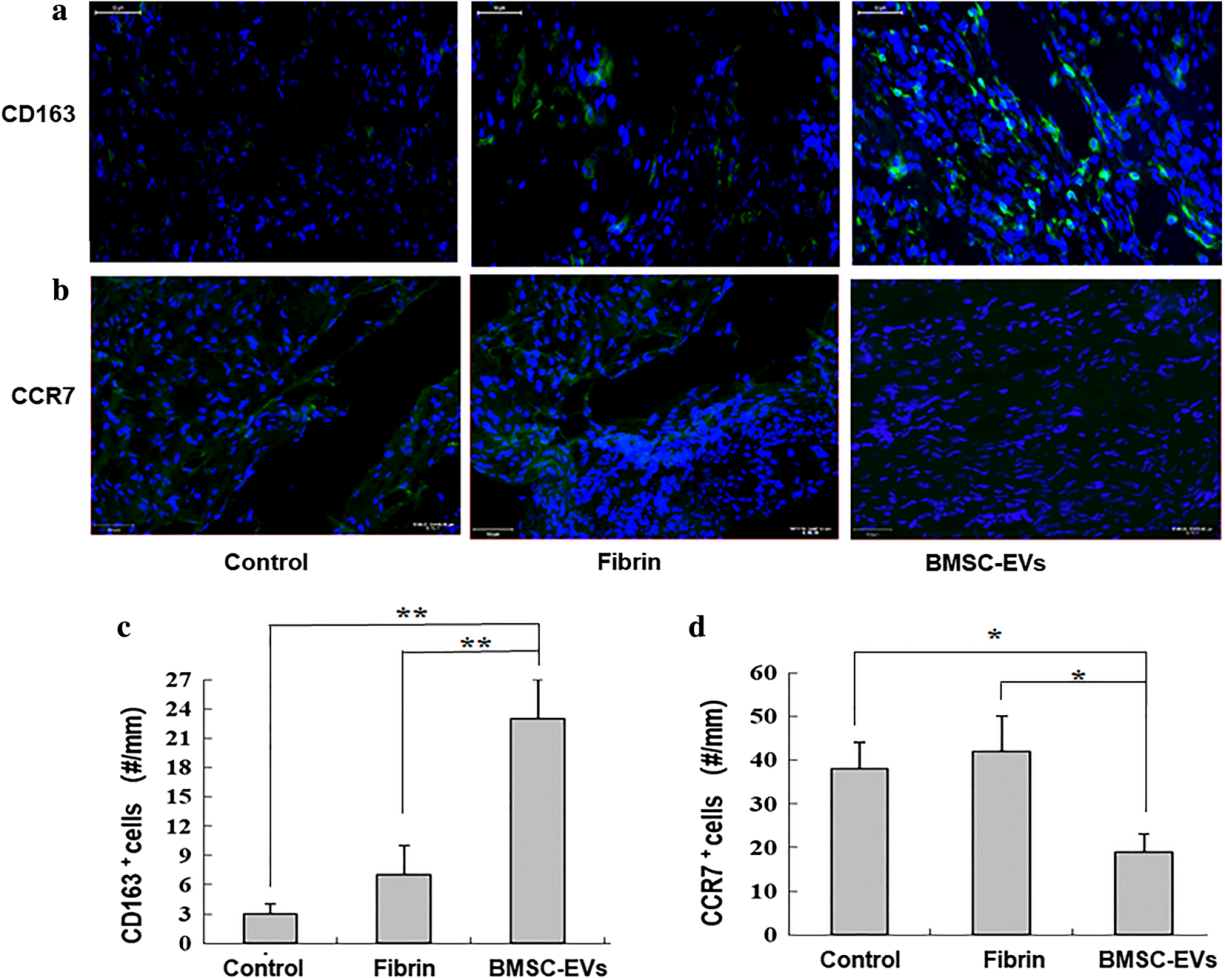
The expressions of **a** CD163+ cells and **b** CCR7+ cells at the repair site were evaluated by immunofluorescence assay. **c** Numbers of CD163+ cells at the repair site (n = 8 donors). **d** Numbers of CCR7+ cells at the repair site (n = 8 donors). Bars: 50 μm, ×200. Data are represented as mean ± SD. *p < 0.05, **p < 0.01

Consistent with the gene expression results, through immunostaining we identified increased numbers of CD163+ cells at the repair site in the BMSC-EVs-treated group (Fig. 6a, c). We also found an accumulation of CCR7+ M1 macrophages in the regions of newly formed tendon tissue in the control and fibrin groups but not in the BMSC-EVs group (Fig. 6b, d), which supports the mRNA expression data.

### Effect of BMSC-EVs on tendon matrix formation

**Fig. 7:**
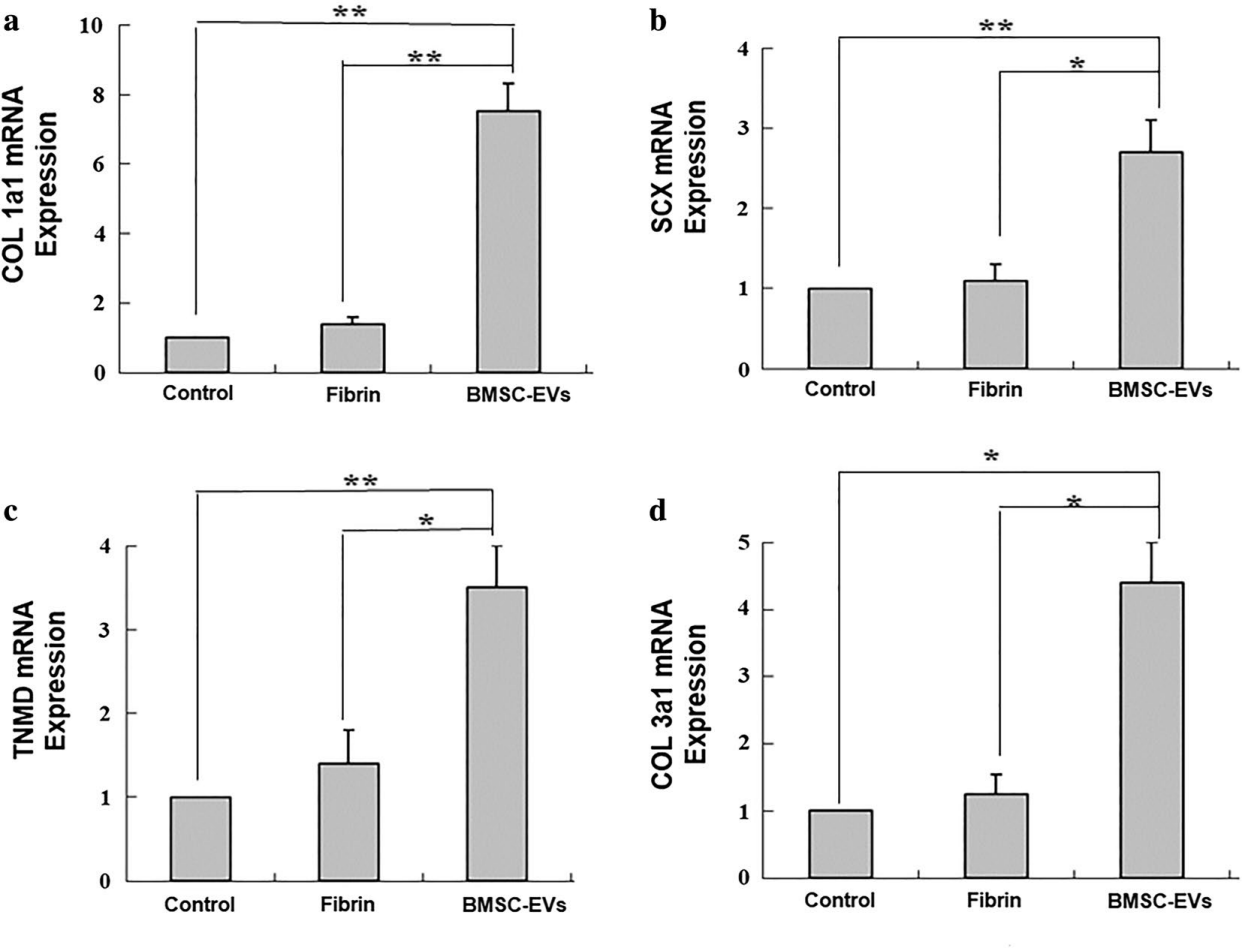
The expression of tenocyte-related genes, **a** COL1a1, **b** SCX, **c** TNMD, and **d** COL3a1 in tendons 14 days after repair (n = 8 donors). Data are represented as mean ± SD. *p < 0.05, **p < 0.01

We found a significantly higher expression of SCX, an early marker for tenogenic differentiation, in the BMSC-EVs groups as compared with the control and fibrin groups (Fig. 7b). Likewise, the expression of TNMD, a mature marker of tenogenic differentiation, was also significantly higher in the BMSC-EVs group compared with the control group (Fig. 7c). For extracellular matrix (ECM) gene expression, COL1a1 (which encodes for type I collagen) and COL3a1 (which encodes type III collagen) were upregulated following BMSC-EVs treatment, with a relatively higher abundance of COL1a1 as compared with COL3a1 (Fig. 7a, d).

### Effect of BMSC-EVs on tenogenesis during tendon healing

**Fig. 8:**
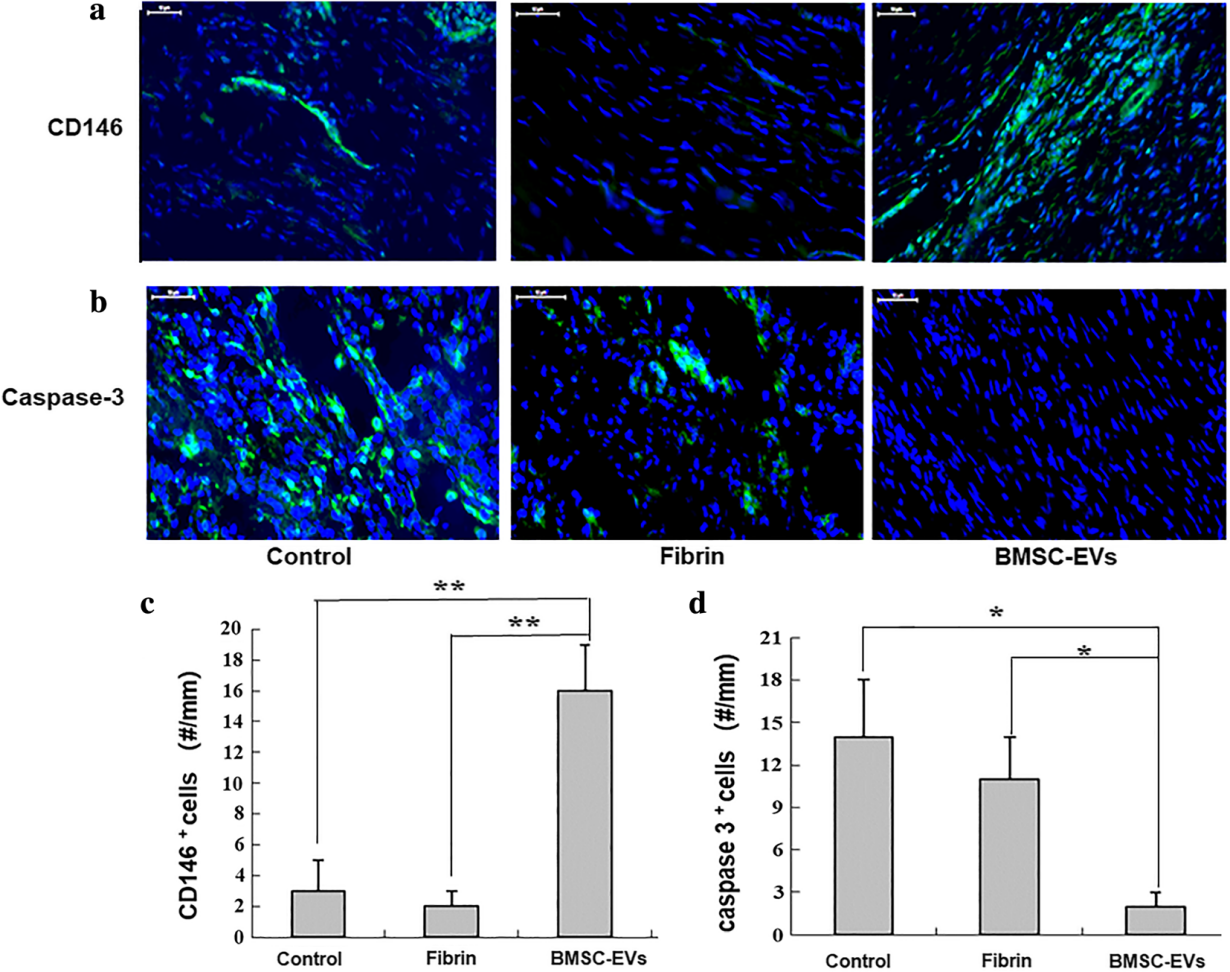
The expressions of **a** CD146+ cells and **b** caspase 3+ cells at the repair site were evaluated by immunofluorescence assay. **c** Numbers of CD146+ cells at the repair site (n = 8 donors). **d** Numbers of caspase 3+ cells at the repair site (n = 8 donors). Bars: 50 μm, ×200. Data are represented as mean ± SD. *p < 0.05, **p < 0.01

CD146+ is a marker of tendon stem cells. At the tissue level, BMSC-EVs treatment induced an accumulation of CD146+ cells at the repair site of treated tendons (Fig. 8a, c). However, no apparent CD146 staining was observed in the control or fibrin groups, suggesting the pro-regenerative effects of BMSC-EVs in tendon repair.

### Effect of BMSC-EVs on tendon cell apoptosis

M1 macrophage activation has been linked to apoptotic cell death at the tendon repair site. Therefore, we tested the potential protective effects of BMSC-EVs against apoptotic cell death during repair. In the control group, cleaved caspase 3 (a marker of apoptotic cells) signals primarily accumulated at the repair site (Fig. 8b, d). Comparatively, cleaved caspase 3 signals were reduced in tendons from the BMSC-EVs group.

### Effect of BMSC-EVs on tendon cell proliferation and collagen type I expression

**Fig. 9:**
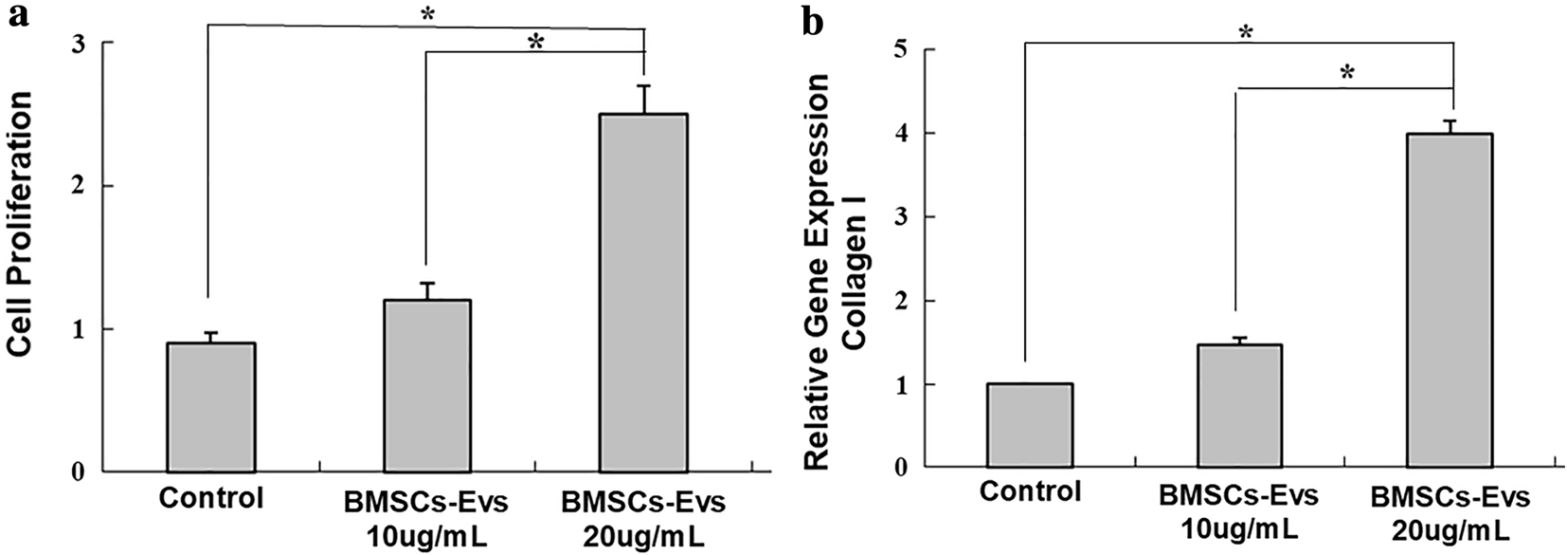
Tendon cell proliferation and collagen type I expression. **a** Treatment of tendon cells with BMSC-EVs (20 μg/mL) increased cell proliferation significantly. **b** The expression of collagen type I, was increased by BMSC-EVs (20 ng/mL). Data are represented as mean ± SD. *p < 0.05

Cells were treated with BMSC-EVs, and viable cells were monitored by CCK-8 assay. Tendon cells exhibited an enhanced proliferative capacity after treatment with BMSC-EVs at 10 and 20 μg/mL concentrations for 3 days (Fig. 9a). However, at concentration of 10 μg/mL, the proliferation of tendon cells was not significantly different from the control. Moreover, the expression of collagen type I, a tenocyte-related gene, were increased by BMSC-EVs (10 and 20 ng/mL) (Fig. 9b), whereas no significant difference was detected at BMSC-EVs (10 ng/mL) group.

## Discussion

Tendon healing progresses through three phases: inflammation, proliferation, and remodeling. The marked inflammatory response that occurs after tendon injury leads to a loss of ECM organization, the formation of scar tissue, and other degenerative events in tendons. Controlling the inflammatory environment after tendon injury is therefore a potential therapeutic target for enhanced healing [23, 24]. Previous studies indicate that early suppression of the inflammatory response through the delivery of MSCs could promote tendon and ligament regeneration to their original state [25, 26]. In this study, we hypothesized that BMSC-derived EVs may play a significant role in regulating inflammation and tendon healing.

We first examined changes in the tendons using H&E staining and Masson’s trichrome staining at 4 weeks after injury. We found that the delivery of BMSC-EVs to the injury site led to a better arrangement of collagen fibers, with fibers more often oriented along the longitudinal axis of the tendon. Masson’s trichrome staining further confirmed the improved tissue quality in the BMSC-EVs-treated group, with an abundance of a collagen-rich ECM in the tendons as compared with those in the other groups.

In many injured tissues, proinflammatory macrophages (M1) promote ECM breakdown, inflammation, and apoptosis [27–29]. However, anti-inflammatory macrophages (M2) often coordinate ECM deposition and tissue repair. Here, we showed a significant reduction in the proportion of CCR7+ M1 cells and an increase in CD163+ M2 macrophages in tendons from the BMSC-EVs group. Furthermore, we found elevated expression of M2 stimulator genes IL-4 and IL-10, and lower levels of inflammatory cytokines (IFNγ, IL-1B, and IL-6). Based on these findings, BMSC-EVs treatment may improve tendon healing by influencing the balance of macrophages and their associated cytokines toward an anti-inflammatory environment. IL13 is a proinflammatory cytokine produced by activated T cells, which can promote the secretion of other inflammatory cytokines and the expression of adhesion molecules. It can regulate collagen stability of fibroblasts. Chronic inflammation induced by IL-13 contributes to the pathogenic scar and fibrosis process by recruiting fibrocytes, which in turn leads to the deposition of excessive ECM that destroy the healing tissue architecture [30]. There was no significant increasement in IL-13 mRNA expression in healing tendon tissue subsequent to BMSCs-EVs treatment in our study, suggesting that BMSCs-EVs may play an important role in improving tendon repair and remodeling after injury.

Based on the changes in the expression of matrix genes involved in tendon healing, our results suggest that BMSC-EVs treatment has a positive effect on repair. We found increased expression of collagen I and III mRNA in healing tendons treated with BMSC-EVs as compared with injured tendons in the control and fibrin groups. Collagen I is the predominant type of collagen in normal tendon tissue, responsible for providing structural and mechanical properties [20]. Type III collagen has been linked to scar formation and inferior tendon mechanical properties after injury [31]. It has been suggested that an optimized collagen type I/III ratio might account for the quality of matrix organization in tendon healing [32]. We found that the relative abundance of COL1a1 was approximately twofold higher than that of COL3a1 in tendons from the BMSC-EVs group. This finding suggests that BMSC-EVs might help to promote the synthesis of ECM components suitable for repair.

We also examined the expression of genes related to tenocyte differentiation. SCX is a basic helix-loop-helix transcription factor involved in tendon development, the deletion of which dramatically disrupts tendon differentiation. SCX is also a distinct marker of tendon progenitors and differentiated cells [33]. In our study, SCX expression was elevated in tendons from the BMSC-EVs group at 2 weeks as compared with the other groups. TNMD is another transcription factor involved in tendon maturation. In the present study, we found a significant increase in TNMD in the BMSC-EVs group over that in the other groups. These results suggest that BMSC-EVs may promote tendon healing by regulating tenogenesis.

Previous studies have revealed that apoptosis plays an important role in regulating tendon regeneration during wound healing [34–36], with higher numbers of apoptotic cells altering the composition of the tendon matrix during repair. Efficient tendon healing should inhibit apoptosis. We found fewer apoptotic cells in the tendons treated with BMSC-EVs, which may suggest that BMSC-EVs can help to prevent the accumulation of apoptotic cells and the subsequent scar tissues that form during early tendon healing.

Previous studies have shown that a reduction in the presence of M2 macrophages and the loss of TNMD expression are associated with fewer CD146-positive cells and more erroneous matrix deposition at the repair site [8, 36]. Furthermore, enriching for CD146-positive cells can lead to improved tendon healing outcomes [6, 8, 37]. Therefore, as a final test, we examined the expression of CD146 in the healing tendons. We found higher proportions of CD146+ cells in the BMSC-EVs group, which suggests the potential for this approach to enhance tendon regeneration. Our findings also demonstrate that regulating the proportion of CD146+ tendon cells may provide an important foundation for the development of a new strategy for tendon healing by encouraging the regenerative capacity of tendon-resident stem/progenitor cells.

Fibrin sealant has been used extensively in all kinds of surgery and research. In this work, an integrated repair was proved effectively by fibrin glue consist of BMSC-EVs in a rat tendon injury model. It could form a stable fibrin polymer after applied to injury site and the window defects of injured tendons were closed with fibrin sealant. Mixing BMSC-EVs in fibrin glue is easy to deliver locally and safe. This method may be a feasible approach for repairing tendon injury in the clinic. Future studies should evaluate the physiological and mechanical properties of the fibrin glue consist of BMSC-EVs for longer period [38, 39].

There were several limitations to the current study. First, the follow-up time points of 2 and 4 weeks in this study may not be long enough for the final analysis, and there may be actual tissue healing status at later time. Study with a longer evaluation period may be needed in future. Second, EVs contain a set of bioactive and tissue trophic molecules such as proteins, nucleic acids, and lipid molecules. Further studies investigating protein or nucleic acids, and which molecular content of the EVs modulate tendon healing are needed to reinforce the results of the current study.

## Conclusions

In summary, we demonstrate that EVs derived from BMSCs can help to improve the quality of tendon healing by modulating macrophage phenotypes, promoting an anti-inflammatory environment, and encouraging the regenerative capacity of tendon-resident stem/progenitor cells. The beneficial effects of BMSC-EVs delivery in tendon healing may offer a new avenue for promoting tendon regeneration. Future studies will explore the potential for this approach to enhance the functional outcomes after tendon repair.

## Data Availability

All data generated or analyzed during this study are included in this manuscript.

## Abbreviations

α-MEM: alpha-modified Eagle’s medium
BCA: bicinchoninic acid
BMSC-EVs: bone marrow mesenchymal stem cells
CCR7: C–C chemokine receptor type 7
COL: collagen
FBS: fetal bovine serum
GAPDH: glyceraldehyde-3-phosphate dehydrogenase
H&E: hematoxylin–eosin
IFNγ: interferon gamma
IL: interleukin
MSCs: mesenchymal stem cells
PBS: phosphate-buffered saline
SCX: scleraxis
SD: standard deviation
TNMD: tenomodulin
TSCs: tendon stem/progenitor cells
